# Ethanolamine and Phosphatidylethanolamine: Partners in Health and Disease

**DOI:** 10.1155/2017/4829180

**Published:** 2017-07-12

**Authors:** Dhaval Patel, Stephan N. Witt

**Affiliations:** ^1^Department of Biochemistry and Molecular Biology, Louisiana State University Health Sciences Center, Shreveport, LA 71130, USA; ^2^Department of Pharmacology, Toxicology and Neuroscience, Louisiana State University Health Sciences Center, Shreveport, LA 71130, USA

## Abstract

Phosphatidylethanolamine (PE) is the second most abundant phospholipid in mammalian cells. PE comprises about 15–25% of the total lipid in mammalian cells; it is enriched in the inner leaflet of membranes, and it is especially abundant in the inner mitochondrial membrane. PE has quite remarkable activities: it is a lipid chaperone that assists in the folding of certain membrane proteins, it is required for the activity of several of the respiratory complexes, and it plays a key role in the initiation of autophagy. In this review, we focus on PE's roles in lipid-induced stress in the endoplasmic reticulum (ER), Parkinson's disease (PD), ferroptosis, and cancer.

## 1. Introduction

The theme of this special issue is bioactive lipids. Bioactive lipids usually are thought to include phosphoinositides, sphingolipids, cholesterol, and eicosanoids, and such molecules have roles in the regulation of cell proliferation, metabolism, organelle function, endocytosis, autophagy, stress responses, apoptosis, and aging. In this issue, we are going to discuss the myriad roles of PE in cells. PE is a lipid chaperone; it is an essential molecule for the synthesis of glycosylphosphatidylinositol-anchored proteins (GPI-AP), which themselves are essential for cell viability, and its covalent attachment to Atg8 triggers autophagosome formation, which is an essential part of autophagy. Very recent findings show the importance of PE to ferroptosis, which is a newly discovered form of cell death, and it is a target of potent anticancer natural products. Here, we will discuss the various aspects of PE activities with respect to health and disease.

## 2. Ethanolamine

### 2.1. Ethanolamine Abundance in Humans

**Figure 1:**
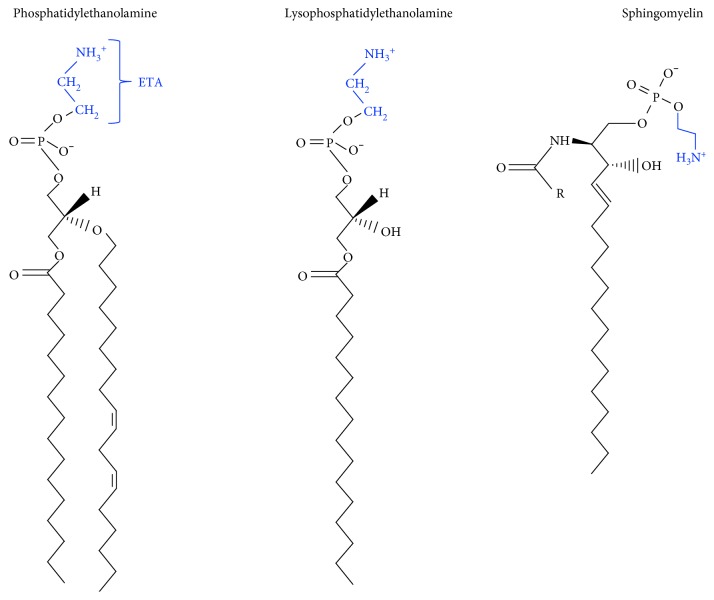
Lipids with a phosphoethanolamine head group.

Essential for life, ethanolamine (H_2_N-CH_2_-CH_2_-OH) occurs in every cell in the human body as the head group of PE (and other lipids) (Figure 1), and it is present as free ethanolamine at varying concentrations in bodily fluids. For example, the concentration of ethanolamine in the blood and breast milk is 2 *μ*M (range 0–12 *μ*M) and 46 *μ*M [1], respectively, whereas the concentration is likely much higher in the gastrointestinal tract due to the breakdown of PE derived from ingested food and the turnover/exfoliation of intestinal epithelial cells. Ethanolamine is a component of GPI-APs, which are essential for viability. Mammals cannot synthesize ethanolamine, and thus it is obtained from the diet as free ethanolamine or in the form of PE, which is degraded by phosphodiesterases to yield glycerol and ethanolamine. Other sources of ethanolamine or phosphoethanolamine in the human body are the degradation of sphingosine phosphate by sphingosine phosphate lyase [2] and the degradation of the endocannabinoid anandamide by the fatty acid amine hydrolase (FAAH) [3].

Several interesting reports regarding the biological effects of ethanolamine have been published: (i) ethanolamine stimulates the rapid growth of mammalian cells in culture; thus, it has been called a growth factor [4–6]. Bovine serum is the source of the ethanolamine found in cell culture media. This growth-stimulatory effect is most likely due to ethanolamine stimulating PE (and phosphatidylcholine, PC) synthesis via the Kennedy pathway (see Phosphatide Precursors Promote Synaptogenesis). (ii) Ethanolamine has a cardioprotective role against ischemia/reperfusion injury via activation of the transcription factor STAT-3 [7]. (iii) Anandamide was shown to reverse the low serum-induced apoptosis of a murine neuroblastoma cell line. Probing the mechanism of this protection, it was discovered that the degradation of anandamide by FAAH was required for protective effect of anandamide; consequently, it was demonstrated that ethanolamine is the compound that protects against the low serum-induced apoptosis [3]. (iv) Ethanolamine and phosphoethanolamine inhibit mitochondrial respiration in a dose-dependent manner by an unknown mechanism [8].

### 2.2. Ethanolamine Interconversion to Other Biomolecules

Plants possess a serine decarboxylase (SDC) that converts serine to ethanolamine (1) [9], whereas humans do not have this capability.

(1) $$\begin{array}{c} \begin{array}{c} SDC\\ Serine\to ethanolamine+{CO}_{2} \end{array} \end{array}$$

Plants can also convert ethanolamine to choline. This is accomplished by three step-wise methylations of phosphoethanolamine to phosphocholine by the enzyme phosphoethanolamine *N*-methyltransferase (P-EAMT) (2) [10].

(2) $$\begin{array}{c} \begin{array}{c} P‐EAMT\\ Phosphoethanolamine\to phosphocholine \end{array} \end{array}$$

**Figure 2:**
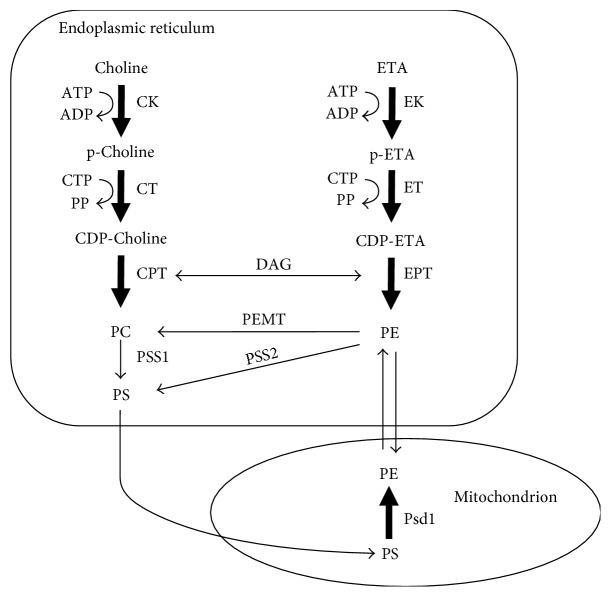
Synthesis of PE via the two major pathways in cells, the Kennedy pathway (ER) and the PSD reaction (mitochondria). The two parallel branches of the Kennedy pathway are the CDP-ethanolamine pathway and the CDP-choline pathway. The four precursors needed for these reactions are choline, ethanolamine, cytosine triphosphate (CTP), and diacylglycerol (DAG). PS is synthesized in the ER via two base-exchange reactions (PSS1 and PSS2). The enzyme PEMT methylates PE to PC. PE is decarboxylated in the inner mitochondrial membrane by PSD (Psd1). CDP: cytidyl diphosphate; CTP: cytidyltriphosphate; DAG: diacylglycerol.

Yeast and humans can also catalyze the step-wise methylation of phosphoethanolamine to phosphocholine; however, the key difference is that in yeast and mammals, the ethanolamine head group of PE (not free phosphoethanolamine) is methylated, yielding PC. Two enzymes carry out this reaction in yeast and one in human cells (PE methyltransferase, PEMT) (Figure 2) [11].

### 2.3. Ethanolamine in the Gut

As a carbon/nitrogen source and a signaling molecule, ethanolamine's dual role is beginning to emerge after decades of research. Gut-associated bacteria such as *Clostridium*, *Listeria*, *Enterococcus*, *Escherichia*, and *Salmonella* [12] contain genes that enable the catabolism of ethanolamine [13]. The catabolism of ethanolamine has been studied in *S. Typhimurium*, a bacterium that contains 17 genes in the *eut* operon that code for proteins involved in the catabolism of ethanolamine [14–17]. Ethanolamine catabolism occurs within a multiprotein compartment called a carboxysome [16]. The ethanolamine ammonia lyase (EutBC) converts ethanolamine into acetaldehyde and ammonia [14, 18]. Acetaldehyde can then be converted to ethanol or, more likely, into acetyl-CoA, which can be used in numerous cell processes (Krebs cycle, glyoxylate bypass, lipid biosynthesis, or other processes) [13]. Acetyl-CoA can also be converted into acetate.

Most strains of *E. coli* and *E. faecalis* are not harmful, whereas these other bacteria listed above are pathogenic. Being able to use ethanolamine as a carbon/nitrogen source likely gives pathogenic bacteria a competitive advantage over other microbial flora. An example is the deadly human pathogen *Escherichia coli* O157:H7 (EHEC), which has genes to sense and utilize ethanolamine [19]. Using ethanolamine as a carbon/nitrogen source gives EHEC a competitive advantage over microbial flora, and, strikingly, ethanolamine activates virulence gene expression in EHEC [19]. Only 1 *μ*M ethanolamine is required to activate virulence gene expression in EHEC, and this concentration is far below the concentration required for ethanolamine to be used as a nitrogen source. The detection of ubiquitous ethanolamine may be a general mechanism by which bacteria sense the intestinal and possibly other host-associated environments [20].

## 3. The Role of PE in Basic Cell Biology

### 3.1. PE Synthesis

#### 3.1.1. PE Synthesis in the ER via the Kennedy Pathway

PE is synthesized in four pathways within two spatially distinct compartments in human cells [21, 22]. Three of the pathways are in the ER while the other is in mitochondria. One of the two major sources of PE is the cytosine diphosphate- (CDP-) ethanolamine or Kennedy pathway, which occurs in the ER [23, 24] (Figure 2). Three sequential enzymatic reactions convert ethanolamine to PE. In the first reaction, ethanolamine is phosphorylated to phosphoethanolamine by the enzyme ethanolamine kinase (EK). In the second reaction, phosphoethanolamine is converted to CDP-ethanolamine by the enzyme CTP:phosphoethanolamine cytidylyltransferase (*Pcyt2*; ET) [25], which uses cytosine triphosphate (CTP) as a cofactor. This reaction is rate limiting. Knocking out both copies of *Pcyt2*^−/−^ in mice causes lethality at 8.5 days in embryonic development (before birth). In contrast, *Pcyt2*^+/−^ mice appear normal and have normal PE levels but have metabolic defects [26]. In the third reaction, CDP-ethanolamine condenses with diacylglycerol to yield PE via the action of integral membrane enzyme 1,2-diacylglycerol ethanolamine phosphotransferase (EPT). Meclizine, which is an over-the-counter drug for motion sickness, is the only inhibitor of the CDP-ethanolamine pathway. Meclizine inhibits ET [27].

PE is also synthesized by two minor routes in the ER. PSS2 (PS synthase 2) catalyzes a calcium-dependent base-exchange reaction whereby the serine group of PS is replaced with ethanolamine [28–30], and lyso-PE acyltransferase converts lysoPE to PE in yeast (and probably humans) [31, 32].

#### 3.1.2. PE Synthesis in Mitochondria by Phosphatidylserine Decarboxylase (PSD)

Mitochondria are the second major source of PE. PSD [33], which is lodged in the inner mitochondrial membrane facing the interstitial space [34, 35], decarboxylates PS to PE (PS→PE + CO_2_) (Figure 2). PSD is a pyruvoyl enzyme that undergoes several processing steps to yield the functional enzyme [33, 36, 37]. Humans express one PSD called PISD, which localizes to mitochondria; whereas, yeast express two: Psd1 localizes to mitochondria and Psd2 localizes to endosomes [38]. One idea is that PS flows into mitochondria from the ER via mitochondrial-associated membranes (MAM) [39], and some PE synthesized via this route is also thought to flow to other compartments via these MAMs. However, this idea was recently challenged by experiments using yeast that showed that the percentage of PS converted to PE in mitochondria by Psd1 was not significantly decreased in yeast mutants that lack both an ER mitochondria encounter structure component and Psd2 [40]. Recent work has demonstrated that PE synthesized in the ER can also transport into the mitochondrial membranes in yeast [41]. The importance of mitochondrial PE synthesis is evident from experiments with transgenic mice. Deleting both copies of the gene for PSD (*Pisd^−/−^*) in mice causes lethality between 8 and 10 days of embryonic development (before birth) [42]. Imaging analysis showed that cells contained aberrantly shaped and fragmented mitochondrion, which was postulated to contribute to cell death. *Pisd^+/−^* mice are viable; however, to compensate for the decreased level of mitochondrial PE, the level and activity of the Kennedy pathway enzyme ET (*Pcyt2*) were significantly upregulated [42]. This compensatory mechanism enables more PE to be synthesized via the Kennedy pathway.

The global deletion of either *Pisd^−/−^* or *Pcyt2^−/−^* causes embryonic lethality in mice. These results demonstrate that the CDP-ethanolamine pathway cannot compensate for eliminating the PSD pathway, and vice versa.

We point out that recent studies have focused on using mass spectroscopy to characterize the mitochondrial lipidome and how it varies in different tissues and different organisms with age [43, 44]. These studies are just the beginning. In the future, we predict an explosion of similar studies that characterize how the mitochondrial lipidome changes in various diseases.

### 3.2. PE Functions

PE is a nonbilayer-forming phospholipid (Figure 1). Its small head group imparts a cone shape to the molecule, and in membranes, the acyl chains of PE impart lateral pressure that can be released by the membrane adopting negative curvature [45]. PE can form a hexagonal phase that is thought to play a role in membrane fusion events [46, 47]. PE, which typically occurs in the inner leaflet of membranes, is abundant in mitochondria. The ethanolamine head group can be covalently modified in numerous ways, as discussed below, and even its acyl side chains are subject to a specific oxidation cell death pathway (see Oxidized PE and Ferroptosis). Overall, PE's numerous activities include, but are not limited to, chaperoning membrane proteins to their folded state [48, 49]; stimulating OXPHOS activity [50, 51]; attaching covalently to the autophagy protein Atg8 [52], which initiates autophagosome formation (see [36] for a review); catalyzing the conversion of prions from the nontoxic to the toxic conformation [53]; being an essential substrate for the synthesis of GPI-APs [54, 55]; and a precursor of other lipids [22]; and it has been implicated in ER stress relating to diabetes and neurodegeneration [56]. Quite stunning recent findings are that PE with arachidonic acyl chains is a target of lipoxygenase, which oxidizes the unsaturated acyl chains into cytotoxic lipid hydroperoxides that promote ferroptosis [57]; PE is the target of a plant natural product that has potent anticancer activity [58], and the mitochondrial protein LACTB is a tumor suppressor that targets PSD for degradation [59]. Because there are many excellent reviews on PE [22, 36, 60], this review focuses on recent findings about PE and lipid-induced ER stress, neurodegeneration, cancer, and ferroptosis.

### 3.3. GPI-Anchor Synthesis and PE

#### 3.3.1. PE Is a Key Substrate for the Synthesis of GPI-APs

**Figure 3:**
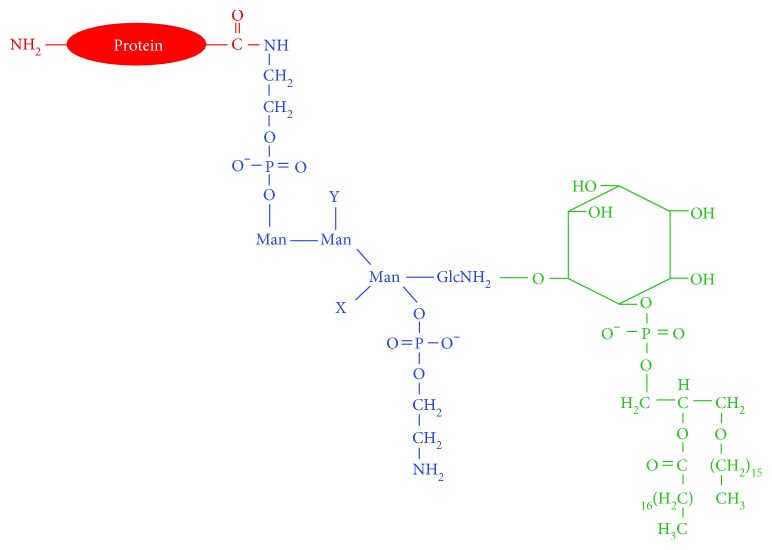
A GPI-AP. A GPI-AP is composed of a lipid tail (green), a conserved glycan core (blue) with attached phosphoethanolamine groups (blue), and the modified protein (red). A phosphoethanolamine moiety, which is extracted from PE, serves as the chemical linker between the GPI anchor and the protein.

A GPI anchor is a glycolipid that is posttranslationally conjugated to the C-terminus of some proteins, and this enables the modified protein to be tethered to the outer leaflet of the plasma membrane [61] (Figure 3). The GPI anchor is found in yeast, protozoa, plants, and humans [62, 63]. The human genome contains approximately 250 GPI-APs, and many of them are essential for the immune response, cell-cell communication, and embryogenesis.

The synthesis of GPI anchors requires PE (Figure 3). The conserved complex glycan core of the GPI anchor is covalently attached in a series of reactions to the inositol ring of phosphatidylinositol (PI). Specifically, over ten steps involving twenty-five different genes are required for the synthesis of a GPI anchor in the ER [64]. Phosphoethanolamine extracted from PE is attached at different sugars that make up the glycan core [65], and in the final step, preformed GPI is attached via a phosphoethanolamine linker (extracted from PE) to the C-terminus of the target protein by a multi-subunit GPI transamidase [66]. Failure to attach or synthesize the anchor causes a rare human disease (see below). Upon entry of a nascent GPI-AP into the Golgi, the lipid chains of the phosphatidylinositol moiety are remodeled to promote association of the GPI-AP with lipid rafts [67], which then transit to the plasma membrane. GPI-APs even recruit other proteins into the lipid rafts [68], for example, in neurons, the PD-associated protein, *α*-synuclein, associates with postsynaptic density protein 90 (PSD-90), which is a GPI-AP [69].

#### 3.3.2. Mutations of Genes in the GPI Anchor Biosynthetic Pathway Cause Paroxysmal Nocturnal Hemoglobinuria

Paroxysmal nocturnal hemoglobinuria (PNH) is a rare, X-linked blood disorder in which red blood cells lack the GPI-APs CD55 and CD59 due to somatic mutations in a gene (phosphatidylinositol glycan class A, PIGA). PIGA is involved in the first step of GPI anchor biosynthesis [70]. Loss of the CD55 and CD59 proteins on the surface of red blood cells results in uncontrolled complement activation and consequently hemolytic anemia, among other problems. A recent case of PNH caused by a germline mutation coupled with a somatic mutation in *PIGT* was reported [71]. *PIGT* codes for a subunit of the transamidase enzyme complex that links the GPI anchor to the target protein. This is a rare case of PNH where the GPI anchor is synthesized but fails to be attached to its target protein. These examples show how mutations impact the synthesis of GPI-APs.

#### 3.3.3. Low PE Causes Inefficient Processing and Maturation of the Protein Gas1 in Yeast

PE is a key substrate for GPI anchor synthesis. If its level decreased in cells for whatever reason, then its lack of availability could adversely affect, like a mutation, the synthesis of GPI anchor proteins. The yeast *Saccharomyces cerevisiae* was used to test whether decreasing the level of PE would affect GPI-anchor protein synthesis. As mentioned above, Psd1 localizes to mitochondria, whereas Psd2 localizes to endosomes. Psd1 accounts for ~70% of the PSD activity in yeast. The double deletion strain *psd1*Δ *psd2*Δ is viable even though it only contains 1 mol% of PE [55]. In these PE-depleted cells, there was a delay in the processing/maturation of the GPI-AP called Gas1 [55]. In contrast, the general secretory pathway was not affected in the PE-depleted cells based on the observation that the protein invertase was secreted into the medium at the same rate as wild-type cells. Thus, PE depletion only affects the vesicular trafficking pathway affecting GPI-APs.

### 3.4. Lipid Disequilibrium and ER Stress

#### 3.4.1. The Unfolded Protein Response: Sensing Disrupted Proteostasis in the ER Lumen

The endoplasmic reticulum compartment, which is surrounded by a membrane bilayer, extends throughout the cell and makes contacts with membranes of organelles such as the nucleus and mitochondria as well as with the plasma membrane. The ER is the early part of the secretory pathway, and it functions to fold and process proteins for secretion and for insertion into membranes. The ER stores calcium and synthesizes lipids and sterols. Up to 60% of the lipids in the cells are contained in the membranes of the ER, and the major phospholipids in the ER are PC and PE. The ER membranes are particularly fluid-like because of the preponderance of PC with unsaturated side chains. The fluidity is required to facilitate the translocation of protein chains in and out of the ER compartment.

If the folding capacity of the ER is exceeded, then toxic unfolded proteins can accumulate in the lumen of the ER, and these unfolded proteins activate a highly conserved stress response composed of three parallel proteotoxic stress-sensing pathways [72, 73]. This response is called the unfolded protein response (UPR). The three stress-sensing pathways in mammalian cells are composed of the activating transcription factor (ATF-6), the inositol-requiring enzyme 1 (IRE-1), and the protein kinase RNA-like ER kinase (PERK). Each of these three proteins contains a lumenal unfolded protein stress-sensing domain, and under normal proteostasis, the stress-sensing domains bind to the chaperone BiP. If unfolded proteins accumulate in the ER, the unfolded proteins preferentially bind to BiP, which releases the stress-sensing domains. For IRE-1 and PERK, the domains dimerize, which triggers association of enzymatic domains on the cytosolic side of the ER, resulting in transautophosphorylation and activation of downstream effectors, that is, genes coding for ER chaperones and lipid synthesis proteins are transcribed and translated. ATF-6 enters the Golgi and is activated therein. In parallel, translation of most mRNA transcripts is downregulated except for those transcripts induced by the response. Usually, upregulating protective chaperones and lipid synthesis enzymes rectify the proteotoxic stress; however, prolonged activation of the UPR can lead to apoptosis. ER stress is thought to contribute to cancer [74], liver disease [75], metabolic disease [76], neurodegeneration [77], and immunity [78, 79].

#### 3.4.2. Lipid-Induced ER Stress

Lipid disequilibrium in the membranes of the ER can also activate the UPR [80]. Studies in this emerging area have revealed that ER-associated sensing/signaling networks that monitor the folding status of the lumenal proteins also monitor the composition of the ER membranes. Recent studies have shown that saturated fatty acids (FA) [81–84], cholesterol [85], an increase in the PC/PE ratio [56, 86], and knocking out desaturases (that add double bonds to PC) [87, 88] trigger the UPR. Whether lipid disequilibrium causes lumenal proteins to unfold or aggregate, which activates the UPR, or the disequilibrium itself activates the UPR (without unfolding proteins), is being investigated. We focus below on some of the studies that have found that lipid disequilibrium activates the UPR which are discussed below.

First, one study explored the cellular defects in obesity and showed that abnormal lipid and calcium metabolism contribute to hepatic ER stress in obesity. The study used livers from lean and obese mice. Specifically, the ER membranes were isolated from hepatocytes and the fatty acid/lipids were determined by MS/MS. A central finding was that the obese ER has a significantly higher ratio of PC-to-PE (PC/PE = 1.97 versus 1.3) than lean ER [56]. This higher PC/PE ratio, an indicator of lipid disequilibrium, was hypothesized to inhibit the calcium transport activity of SERCA, resulting in altered calcium homeostasis. Confirming this hypothesis, inhibition of PC synthesis decreased PC and increased PE to yield a PC/PE ratio of 1.3, which is equivalent to this ratio in lean ER. Moreover, inhibiting PC synthesis improved calcium transport. The overall conclusion was that obesity leads to lipid disequilibrium, which alters calcium homeostasis leading to ER stress and chronic activation of the UPR. Second, digging into the mechanism by which changes in lipid saturation activate the UPR, it was shown that mutant mammalian ER stress sensors, IREa and PERK, which lack their lumenal unfolded protein stress-sensing domain, nevertheless retain sensitivity of their enzymatic domains to increases in lipid saturation [89]. The membrane-spanning domains of IREa and PERK were, however, required to maintain sensitivity to changes in lipid saturation. IRE1 and PERK are thus lipid sensors that can act independently from the conventional sensing of proteotoxic stress [84, 89]. Third, another group found that UPR can be activated via lipid disequilibrium without disturbed proteostasis [88]. *C. elegans* that lack the subunit, *mdt-15*, of the Mediator, which is a highly conserved transcriptional regulator, have defects in reproduction, mobility, and a shortened lifespan. Worms depleted in *mdt-15* have lower levels of phospholipid desaturation, especially with respect to PC, and that such worms have a constitutively activated UPR. *mdt-15* controls the expression of three FA desaturases (*fat-5*, *fat-6*, and *fat-7*); *fat-6* and *fat-7* are referred to together as “stearoyl-CoA-desaturases” (SCDs) [90, 91]. Knockdown of *SCD*s increases the level of saturated FAs in the ER and activates the UPR without triggering proteotoxic stress [87, 88]. Significantly, no synthetic lethality occurred when *SCD* was knocked down in cells that also had mutations of the UPR genes, which are known to cause protein misfolding. This elegant study demonstrated that the UPR is induced by an imbalance between saturation and unsaturation of ER lipids.

## 4. The Role of PE in Human Disease

### 4.1. PD, *α*-Synuclein, and PE

#### 4.1.1. PD and *α*-Synuclein

PD is the most common neurodegenerative movement disorder [92]. There are two forms of the disease. Sporadic or idiopathic PD occurs late in life, and there are no associated genetic defects. The biggest risk factor for sporadic PD is age. Familial or early onset PD occurs early in life, and such patients have mutations in one of several genes. In PD, dopaminergic (DA) neurons in a region of the brain called the *substantia nigra pars compacta* (SNc) progressively die off with age, which leads to the classic symptoms of resting tremor, disturbances of gait and balance, and postural instability. By the time a person experiences these symptoms, it is thought that as much as 80% of the DA neurons have died. There are no treatments to regenerate the neurons. The vast majority of patients receive dopamine replacement therapy (L-DOPA). At the cellular level, the affected neurons often contain proteinaceous inclusion bodies called Lewy bodies (LB) [93]. The principal component of LBs is the protein *α*-synuclein [94]. The discovery of *α*-synuclein in Lewy bodies was preceded by the discovery that a missense mutation of the *α*-synuclein gene, *SNCA*, causes early onset PD [95]. The discoveries that both missense mutations of *SNCA* and multiplications [96] of the *SNCA* locus cause early onset PD and that wild-type *α*-synuclein is the principal component of LBs have led to an explosion of research into the structure and function of this mysterious protein. Here, we discuss sporadic PD.

#### 4.1.2. PD and Possible Deficits of PE

PD is a disease of aging [97]. We are interested in the lipidome of the brain, how it changes with age, and whether the changes are related to the onset of PD or merely an epiphenomenon. Data from many sources indicate that low PE can occur with age and may be a factor in PD. First, PE in the SNc of PD patients is significantly lower compared with control subjects [98]. Second, phosphoethanolamine levels are significantly lower in the midbrain of early PD patients but not in the advanced patients compared with control subjects, according to a recent imaging study [99]. Additionally, phosphoethanolamine is also significantly lower in the cerebrospinal fluid of PD patients compared with controls [100]. Third, in mice, PE (o-32:1) significantly decreases (3.2-fold decrease) in aged brain mitochondria (78 weeks) compared to young brain mitochondria [43]. Fourth, the activities of Kennedy pathway enzymes phosphoethanolamine cytidylyltransferase, phosphocholine cytidylyltransferase, and PS synthase are significantly elevated in the *substantia nigra* of PD patients compared with controls [101]. Increases in the activities of these enzymes are a likely compensatory mechanism in response to low PE/PC (see PE Synthesis). Fifth, ethanolamine significantly protects against *α*-synuclein-induced degeneration of dopaminergic neurons in *C. elegans* [86]. Sixth, PE decreases by as much as 50% with age in genetically identical male mice but not, surprisingly, in female mice [102]. Curiously, *α*-synuclein expression in the nervous system blocks the decrease in PE with age in male mice. Given that brain PE can decrease with age, an abnormally high PC/PE ratio will likely ensue. Consequences of low PE are chronic lipid-induced ER stress [86], inefficient processing of GPI-APs [55], and possibly impaired autophagy because PE is covalently attached to Atg8, which triggers autophagosome formation [36, 52].

#### 4.1.3. Low PE in Yeast and Worms Is Synthetically Toxic with *α*-Synuclein

Whether low PE affects the trafficking of *α*-synuclein through cells was recently addressed using yeast and worms [86]. In yeast, the Psd1 deletion strain, *psd1*Δ, is viable even though the cells have ~50% less PE than the wild-type cells. *psd1*Δ cells expressing *α*-synuclein die from a combination of ER stress, inability to process GPI-APs, and a build-up of *α*-synuclein [86]. Empty vector (EV) *psd1*Δ cells displayed intense ER stress in a *β*-gal assay stress assay, whereas *psd1*Δ cells expressing *α*-synuclein displayed the same level of stress as EV cells; thus, the ER stress in *psd1*Δ cells is due to low PE (not *α*-synuclein). Supplementing yeast cells with ethanolamine increased the level of PE (via the Kennedy pathway, Figure 2()), abolished ER stress and a-synuclein foci, decreased the level of *α*-synuclein, and restored growth.

To further probe the effects of lipid dyshomeostasis on the formation of *α*-syn foci in yeast cells, the lipid metabolism mutants *cho1*Δ, *cho2*Δ, *opi3*Δ, and *ino2*Δ were also tested for *α*-synuclein foci and the PC% and PE% were determined. Cho1 catalyzes the reaction of CDP-diaclyglycerol and L-serine to yield PS, which is the substrate for Psd. Cho2 and Opi3 are methylases that convert PE to PC. Cho2 catalyzes the first methylation, whereas Opi3 catalyzes the second and third methylations. Ino2 is a transcription factor that regulates phospholipid biosynthesis. *α*-synuclein-GFP formed foci in *cho1*Δ, *cho2*Δ, *ino2*Δ, *opi3*Δ, and *psd1*Δ cells, whereas no foci formed in wild-type cells or *psd1*Δ cells treated with ETA or choline. An *x*, *y* plot of PE%, PC% data points revealed that when PC% + 1.38 PE% > 23.7%, *α*-synuclein is soluble, whereas when PC% + 1.38 PE% ≤ 23.7%, *α*-synuclein forms foci. One might ask, how can choline rescue the low PE phenotype of the *psd1*Δ mutant or of worms with Psd1 knocked down by RNAi [86, 103]? One possibility is that choline is converted to PC by the Kennedy pathway, to PS (via base-exchange with serine), and to PE (via Psd1) (Figure 2()). Likewise, ethanolamine can rescue cells with low PC because added ethanolamine is converted to PE via the Kennedy pathway and PE is methylated to PC by the enzyme PEMT (Figure 2()).

In parallel experiments, the worm ortholog of PSD (*psd-1*) was knocked down using RNAi in DA neurons that express human *α*-synuclein [86, 104, 105]. *α*-syn/*psd-1* RNAi worms displayed significantly more neurodegeneration at day 7 after hatching than *α*-syn/EV control worms. Thus, similar to yeast, low PE (due to knocking down *psd-1*) is synthetically toxic with *α*-synuclein. ETA supplementation over several days rescued neurodegeneration in *α*-synuclein/*psd-1* worms. Strikingly, ETA also rescued the age-dependent neurodegeneration in *α*-synuclein/EV control worms, which was unexpected because such worms should have normal levels of PE. Collectively, ETA rescues *α*-synuclein–induced neurodegeneration with or without the depletion of *psd-1*. Such a finding suggested that PE declines with age in the worm DA neurons. We pointed out that PE may decline with age in the human brain with age in PD and Possible Deficits of PE.

#### 4.1.4. Model for How Low PE Induces the Aggregation of *α*-Synuclein

**Figure 4:**
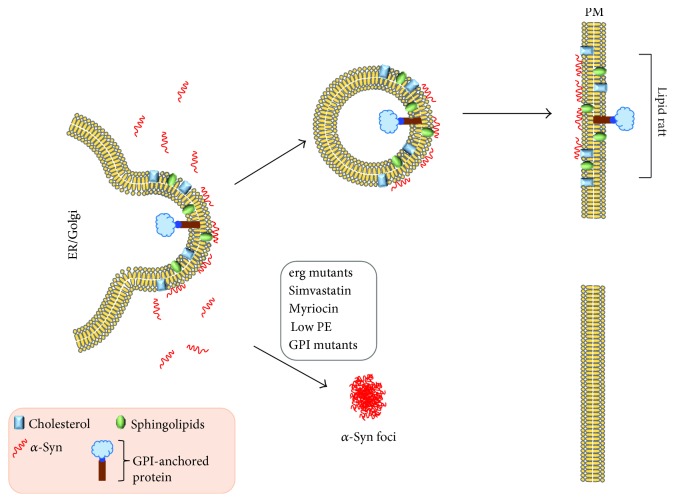
Model for the aggregation of the PD-associated protein *α*-synuclein. Low PE generates lipid-induced ER stress and disrupts the synthesis and vesicular trafficking of GPI-APs. The disruption of GPI-APs via low PE or mutations in a GPI anchor gene triggers *α*-synuclein to form cytoplasmic foci. Similar foci form when the synthesis of ergosterol/cholesterol or sphingolipids is inhibited pharmacologically or via mutation. Ergosterol/cholesterol and sphingolipids make up lipid rafts and GPI-APs partition into lipid rafts. Disrupting GPI-anchor protein synthesis or lipid raft composition results in the formation of *α*-synuclein foci. Modified from [86].

A model for how low PE affects *α*-synuclein-expressing cells is shown in Figure 4() [86]. The model synthesizes results from numerous labs [55, 68, 86]. Low PE in *psd1*Δ cells generates intense ER stress [86], and low PE specifically inhibits the vesicular pathway that traffics GPI-APs to the plasma membrane [55]. The combination of lipid-induced ER stress and inefficient trafficking of GPI-APs in *psd1*Δ cells causes the *α*-synuclein protein level to increase, which triggers the formation of cytoplasmic foci of synuclein [86]. Such foci also form in *α*-synuclein-expressing cells when sphingolipid [106] or ergosterol [107] synthesis is inhibited. Strikingly, in mammalian cells, depleting cholesterol with *β*-methylcyclodextrin also impedes the vesicular trafficking of *α*-synuclein [69], indicating that lipid rafts mediate the trafficking of *α*-synuclein to the plasma membrane. These data show that the integrity of the lipid rafts is essential for the intracellular trafficking of both GPI-APs and *α*-synuclein. Perturbations of this pathway shunt *α*-synuclein into dead-end vesicles that accumulate in the cytoplasm. Overall, the proposed model contains features such as ER stress [108, 109] and the formation of *α*-synuclein deposits [110] that occur in mammalian PD models. In the context of this model, supplemental ethanolamine rescues *α*-synuclein toxicity because it converts to PE via the Kennedy pathway (Figure 2()), and increasing PE improves the processing of GPI-APs [55], decreases ER stress [86], and increases autophagic flux [111] (because autophagy depends on PE for formation of the autophagosome).

### 4.2. Phosphatide Precursors Promote Synaptogenesis

The use of phosphatide (phospholipid) precursors to promote synaptogenesis to improve memory in Alzheimer's disease patients is an intriguing area of research [112, 113], and this topic is germane to the discussion of ethanolamine, PE, and PD (see above). Choline, a pyrimidine (uridine), and polyunsaturated fatty acids (PUFAs) (DHA, docosahexaenoic acid) are three precursors required for optimal stimulation of PC synthesis via the CDP-choline pathway. These compounds readily cross the blood-brain barrier to stimulate the synthesis of PC, resulting in enhanced synaptic activity, increased numbers of dendritic spines, increased release of neurotransmitters, and improvement in cognition (reviewed in [112, 113]). Uridine upon entering the brain is converted to CTP by CTP synthase [106]. These three precursors plus certain vitamins are being tested in clinical trials in Alzheimer's disease, where synapse loss is a serious problem.

A synapse, which is a structure that enables cell-to-cell communication, is composed of a presynaptic terminal (from an axon of one neuron), a synaptic cleft, and a postsynaptic membrane (attached to a dendrite of cell body of another neuron). When an impulse reaches the presynaptic terminal, synaptic vesicles merge with the presynaptic membrane, releasing neurotransmitter into the synaptic cleft. The neurotransmitter binds to receptors on the postsynaptic membrane, which in turn triggers the postsynaptic neuron to send an impulse to the next synapse. A dendritic spine is a membranous protrusion from the dendrites of neurons.

Wurtman pioneered nutritional supplements as a way to promote synaptogenesis. The rationale of such supplements is that, “the brain is unusual among organs in the extent to which the rates of its most characteristic biochemical reactions are controlled not by the amount or activity of a key enzyme, but rather by the extent to which that enzyme is saturated with its substrate, which usually is both a nutrient and a precursor for a physiologically active reaction product [112].” Support for this idea is that the rate at which neurons synthesize and release a variety of neurotransmitters (serotonin, acetylcholine, and dopamine) is accelerated when the precursors (tryptophan, choline, and tyrosine) of these neurotransmitters are administered [114–116]. Wurtman and colleagues have published numerous studies that have demonstrated that administering the three circulating nutritional precursors—uridine, DHA, and choline—to animals (rats, gerbils) significantly increased the levels of PC and PE and other lipids (sphingomyelin (SM), PS, and PI) in the brain [117–120] and that this treatment promotes neurite outgrowth, increases synaptic proteins and phospholipids, and increases potassium-evoked dopamine release in aged rats [120–122]. These three nutritional precursors increase PE and PS via the reactions in Figure 2. Increasing PC increases SM levels because PC is one of the two substrates for SM synthesis [123]. Likewise, uridine converts to CTP in the brain which increases the level of CDP-diacylglycerol, which is a substrate required for PI synthesis [124].

How do these nutritional precursors relate to Alzheimer's disease? It is thought that in Alzheimer's disease, amyloid plaques damage dendritic spines and synapses and prevent new synapses from forming [125, 126]. The idea is that supplementing an organism with these three precursors will stimulate the synthesis of PC (and PE and other lipids) in the brain, which thereby promotes dendritic spine and synapse formation. This treatment might reverse or slow down damage from amyloid plaques.

We suggest that in addition to stimulating synaptogenesis that choline, uridine, and DHA create a “perfect storm,” in the good sense, they also promote efficient processing of GPI-APs, decrease lipid-induced ER stress, and increase autophagic flux. It is tempting to speculate that stimulating these processes may decrease the accumulation of cytotoxic misfolded/unfolded proteins such as aggregated *α*-synuclein.

### 4.3. PE and Cancer

#### 4.3.1. OPA Is a Natural Product with Potent Anticancer Activity

**Figure 5:**
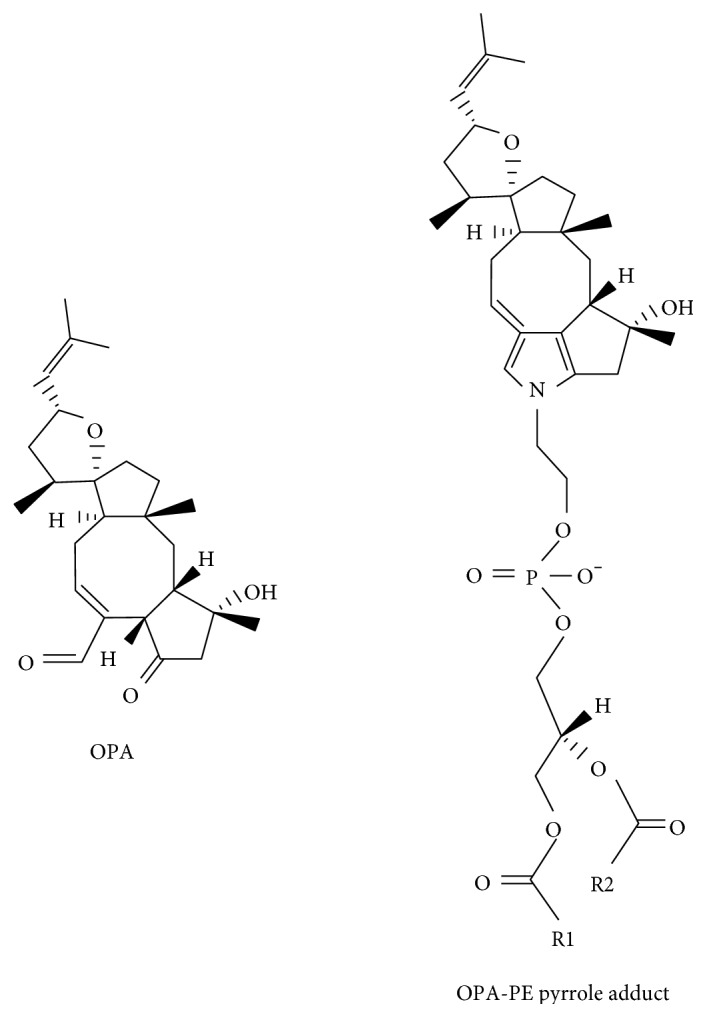
Ophiobolin A (OPA) and its cytotoxic adduct with PE (OPA-PE). PE is abundant in the inner leaflet of most cell membranes. However, by unknown mechanisms, some cancer cells flip PE from the inner leaflet to the outer leaflet. OPA has been proposed to react with the PE in the outer leaflet generating a cytotoxic species that causes leaky membranes and that eventually kills the cells.

OPA is a sesterterpenoid secondary metabolite isolated from pathogenic fungi of the *Bipolaris* genus [127] (Figure 5). Fungi that synthesize this metabolite cause brown spot lesions on crops such as maize, rice, and sorghum. OPA has been reported to promote the leakage of electrolytes and glucose from maize seedling roots [128], and it covalently modifies calmodulin at a lysine residue [129]. OPA has potent anticancer activity [130], and it kills glioblastoma cells [131], which is of great clinical importance because glioblastomas are aggressive and resistant to most drugs. Most anticancer drugs induce apoptosis, and for unknown reasons, glioblastomas do not die via apoptosis. OPA may be effective against glioblastoma because it induces paraptosis, which is a form of programmed cell death that is morphologically and biochemically distinct from apoptosis [132]. Paraptosis is defined by vacuolization that begins with the enlargement of mitochondria and the ER, possibly due to disrupted potassium ion homeostasis. Because of OPA's effectiveness against glioblastoma and because its biochemical target is not known, investigators recently used a powerful genetic screen to search for the cellular target of OPA, as described below.

#### 4.3.2. PE Is the Target of OPA

A loss-of-function genetic screen using human near-haploid KBM7 cells [133, 134] was conducted to search for the target of OPA [58]. KBM7 cells are a myeloid cancer cell line. The cells are infected with a virus that makes insertions into the genome that result in gene inactivation. If an essential gene is knocked out, the cells will die. If a nonessential gene is knocked out, the cell will be viable and form colonies if inactivation of the gene causes resistance to OPA. Using this strategy and sophisticated bioinformatics and statistical techniques, it was discovered that KBM7 cells are resistant to OPA only when *EK* or *ET* or *EPT* is inactivated. Strikingly, of the thousands of genes in the human genome, these three genes code for the three enzymes of the CDP-ethanolamine pathway (Figure 2). Knocking down *ET* (*PCYT2*) in three different cell lines decreased the level of PE and made cells resistant to OPA. Further characterization revealed that OPA reacts with the amino head group of PE to form a cytotoxic PE derivative with a bulky pyrrole-like head [58] (Figure 5). On the basis of these results, it was hypothesized that PE is the target of OPA and that PE-OPA derivatives kill cells by creating leaky membranes. This was tested using synthetic liposomes (with varying contents of PE) loaded with a fluorescent dye. OPA induced leakiness of the liposomes in a dose- and PE-dependent manner, and OPA failed to induce leakiness in liposomes devoid of PE. The authors concluded that PE is the target of OPA (Figure 5). This unexpected finding adds a new twist to the chemistry of PE.

#### 4.3.3. Exposure of PE and PS on the Surface of Cancer Cells

**Figure 6:**
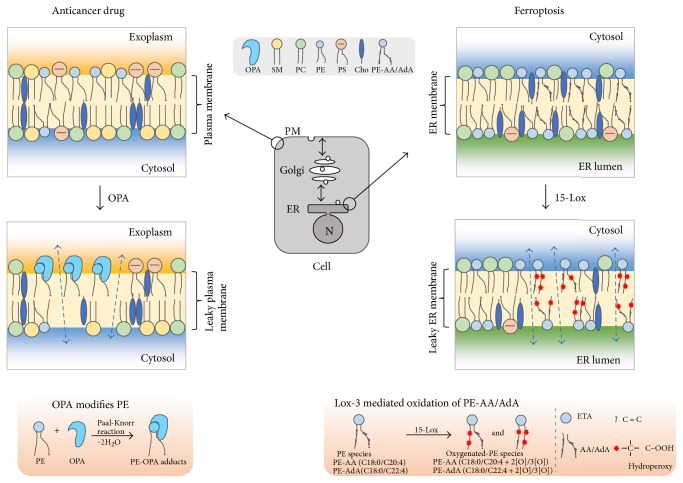
PE in cancer and ferroptosis. Left panel: PE and PS transfer to the outer leaflet of the plasma membrane in some cancers. OPA reacts with surface-exposed PE to yield a cytotoxic adduct that creates leaky membranes (arrows) that kills cells. Right panel: Lipoxygenase (15-Lox) oxidizes PE in the membranes of the ER. The oxidation requires that PE contains polyunsaturated acyl chains like arachidonic acid (A) or adrenic acid (AdA). The end product is doubly and triply oxidized hydroxyperoxide PE species that mediate cell death.

PE and PS are asymmetrically distributed in mammalian cells, in that, each of these lipids is predominantly in the inner leaflet of the plasma membrane. Therefore, how is it that cancer cells have PE in the outer leaflet of their plasma membrane? First, we point out that PE comprises 5% of the phospholipid content of the outer leaflet of erythrocytes [135], and oxidizing agents increase the amount of PE in the external leaflet [136]. Second, PE as well as PS were shown to be exposed in the outer leaflet of the plasma membrane of cytotoxic T cells undergoing the early stages of apoptosis [137] as well as cells exposed to irradiation [138]. Third, one study showed an increase in the exposure of PE on the surface tumor vasculature endothelium [139]. Another study that screened fifteen different cancer cell lines for surface PE using a fluorescent duramycin analog detected low levels of surface PE in twelve of the cancer cell lines and high surface PE in three multiple myeloma cell lines [140]. Fourth, surface-exposed PS and PE in synthetic liposomes synergistically enhance the pore-forming activity of a peptide with anticancer properties [141]. Perhaps flippase activity is disrupted in some cancer cells, and this could result in the flipping of PE to the outer leaflet of the plasma membrane. Chidley and coworkers proposed that OPA reacts with surface-exposed PE, and that the bulky OPA-PE adducts disrupt the cell membrane, creating leakiness that kills the cancer cells (Figure 6).

#### 4.3.4. LACTB Is a Tumor Suppressor

A unique screen for tumor suppressors was recently conducted [59], and the results are germane to this review. Keckesova et al. reasoned that while the incidences of breast, lung, and colon cancers are quite high, cancers of the heart, skeletal muscle, and brain are exceedingly rare, almost unheard of. “Cancer-resistant” cell types, such as cardiomyocytes, are nonproliferative, terminally differentiated, and use oxidative phosphorylation over glycolysis for the production of ATP; whereas, cancer cells are proliferative, relatively undifferentiated, and use aerobic glycolysis instead of oxidative phosphorylation for the generation of ATP. By using glycolysis as the main source of energy, cancer cells have a plethora of three-carbon compound metabolites from which the building blocks (proteins, lipids, and DNA) for new cells can be made. The clever idea was that factors that induce or maintain cells in a nonproliferative, differentiated state that uses oxidative phosphorylation could function as tumor suppressors if introduced into the neoplastic state [59]. Consequently, gene expression microarray analysis was performed to identify mRNAs that were upregulated in differentiated muscle cells from mice and humans versus undifferentiated, actively cycling cells. This analysis led to the discovery that *LACTB* overexpression had the most potent inhibitory effect on proliferation.

#### 4.3.5. The LACTB Protein Is a Highly Conserved Mitochondrial Protein; LACTB Is an Obesity Gene

Previous studies have shown that LACTB is an evolutionarily conserved mitochondrial protein related to gram-negative bacterial penicillin-binding/B-lactamase proteins [142, 143]. The protein is expressed in the heart, liver, and skeletal muscle [142, 144]. A serine protease confined to the intermembrane space of mitochondria, LACTB polymerizes into long filaments that may promote intramitochondrial membrane organization [145]. Before the Kechesova work, whether LACTB also functions as a protease, in addition to its structural role in mitochondria, was unknown. Another interesting feature is that *LACTB* is a bona fide obesity gene. *LACTB* transgenic mice displayed a 20% increase in fat-mass-to-lean-mass ratio compared to wild-type control mice [146]. The combined results are consistent with LACTB globally influencing metabolism.

#### 4.3.6. LACTB Is a Mitochondrial Protein That Is Downregulated in Many Cancer Cell Lines

Analysis of LACTB protein level in 18 breast cancer cell lines revealed that LACTB expression was downregulated (but never completely absent) in 15 of the 18 cell lines tested [59]. Although the MCF7-RAS breast cancer line showed LACTB levels similar to that in nontumorigenic cell lines, this cell line was found to have a R469K mutation in the LACTB gene. The position of this amino acid substitution in the LACTB may inactivate the protein given that the substitution is close to three important catalytic and/or substrate-docking domains. Overexpressing LACTB in already formed tumors of MCF7-RAS, HMLER, and HCC1806 dramatically decreased the size of or even eliminated the tumors. Knocking down LACTB expression via gene silencing in nontumorigenic HME cells caused a two-fold decrease in growth rate compared to control HME cells. The HME cells with LACTB knockdown failed to form tumors when the cells were implanted in nonobese diabetic/severe combined immunodeficiency (NOD/SCID) mice. On the other hand, because knockdown of a tumor suppressor gene often must be accompanied by expression of an oncogene for transformation to occur, Keckesova knocked down LACTB in HME cells containing the oncogene *HRAS^G12V^*. Such cells transplanted into NOD/SCID mice formed tumors 6 weeks after injection, whereas *HRAS^G12V^* cells failed to form tumors even 12 weeks after injection. For transformation to occur, LACTB knockdown must be accompanied by an oncogene.

#### 4.3.7. LACTB Degrades PISD, the Supplier of PE to Mitochondria

Analysis of mitochondrial lipids isolated from tumorigenic cells in which LACTB was induced for 24 h revealed that PE and LPE were decreased by 30–50% in MCF7-RAS cells but not in the nontumorigenic HME control cells. Supplementing the tissue culture medium of LACTB-induced MCF7-RAS cancer cells with 20 *μ*M LPE (but not PE) increased proliferation, in essence partially reversing the growth inhibitory effect of LACTB expression. Collectively, decreased levels of LPE and/or PE mediate a substantial part of the LACTB-induced negative effects on MCF7-RAS cells.

Given the decreased levels of PE and LPE in LACTB-induced MCF7-RAS cells, experiments were conducted to ascertain the status of mitochondrial PISD, the enzyme that converts PS to PE in mitochondria (Figure 2). Indeed, overexpressing LACTB in MCF7-RAS cells decreased the level of the PISD protein by 60–90% compared to the same cells without LACTB induced. Overall, LACTB decreases PISD and hence PE/LPE in some but not all cancers and fails to do this in nontumorigenic cell lines. It is presumed but not proven that the protease activity of LACTB is responsible for the decrease in the level of PISD. The signaling pathways that enable LACTB to suppress proliferation of some cancers but not others will be the subject of future investigations.

### 4.4. Oxidized PE and Ferroptosis

#### 4.4.1. Ferroptosis, a Newly Discovered Form of Cell Death: Identification of Inhibitors

Ferroptosis is a newly discovered form of cell death that is distinct morphologically, biochemically, and genetically from apoptosis, autophagy, and necrosis [147, 148]. Ferroptosis was only characterized and named in 2012; thus many of the mechanistic details regarding this form of cell death are still being unraveled. The concept of ferroptosis came out of studies of compounds that kill RAS mutant tumor cells. Up to 30% of all cancers have mutations in RAS, which are a family of small GTPases (HRAS, NRAS, and KRAS) that regulate cell growth, adhesion, differentiation, migration, and survival [149]. Pancreatic cancers often have mutations in KRAS, and few chemotherapeutics are effective against cells that harbor such mutations. The discovery that the compounds erastin and RSL3 are potent killers of RAS mutant cell lines led to the discovery and elucidation of this new cell death pathway [150]. Erastin- and RSL3-induced cell death were characterized by high levels of intracellular reactive oxygen species (ROS), and iron chelators or genetic knockdown of iron transporters abolished the drug-induced ROS. No evidence of apoptosis (cytochrome c release, caspase activation, and chromatin condensation) was observed. Because iron chelators rescued erastin- and RSL-3-induced cell death, it was hypothesized that ROS was being generated in an iron-dependent reaction, possibly the Fenton reaction. The name ferroptosis came about because of the role of iron in this cell death pathway. Erastin and RSL-3 were found to disrupt redox homeostasis, that is, erastin inhibits the import of cysteine via an antiporter system called x^−^_C_ and RSL-3 inhibits glutathione peroxidase 4 (GPX4), which catalyzes the reduction of phospholipid hydroperoxides and neutral lipid hydroperoxides to their hydroxyl forms [150].

#### 4.4.2. Acyl-CoA Synthetase Long-Chain Family Member 4 (ACSL4) Is an Essential Component of the Ferroptosis Circuitry

A recent study using two approaches—a genome-wide CRISPR-based genetic screen and a microarray analysis of ferroptosis-resistant cells lines—found that the gene acyl-CoA synthetase long-chain family member 4 (ACSL4) is an essential component of the ferroptosis circuitry [151]. This means that *Acsl4* KO cells were resistant to RSL-3-induced ferroptosis. ACLS4 converts free long polyunsaturated *ω*^6^ fatty acids, like arachidonic acid (AA) and adrenic acid (AdA), into acyl-CoA esters. The notion was that polyunsaturated acyl chains may be the target of the iron-dependent ROS that is triggered in ferroptosis. One way to monitor lipid peroxidation is with the peroxidation-sensitive dye BODIPY 581/591 C11. When the C11 chain is oxidized, the fluorescence emission wavelength of the dye changes. *Acsl4* KO cells, which have low levels of polyunsaturated *ω*^6^ fatty acids, were resistant to RSL-3-induced peroxidation of this dye. An analysis of the oxidized lipid species in *Acls4* WT and KO cells treated with RSL-3 showed that the *Acls4-*deficient cells had significantly lower levels of PE species containing doubly and triply oxidized AA and AdA lipids (all-cis-7,10,13,16-docosatetraenoic acid) side chains. Thiazolidinediones are pharmacologic inhibitors of ACLS4 (but not other ACLS isoforms), and these drugs indeed inhibit ferroptosis, which further confirms ACLS4 as a node in the ferroptotic circuitry. An intriguing finding was that—for a variety of cell types—knocking down *Gpx4* results in cell death, whereas *Acls4* and *Gpx4* double knockout cells are viable and proliferate normally. The inference of this finding is that decreasing the amount of long polyunsaturated *ω*^6^ fatty acids prevents the formation of cytotoxic, ferroptosis-inducing oxidized PE species, which obviates the need for GPX4.

#### 4.4.3. Ferroptosis Occurs in the ER; Hydroperoxy-PE Species Mediate Cell Death

A parallel study, using quantitative redox lipidomics, reverse genetics, bioinformatics, and systems biology, showed that oxidation of polyunsaturated lipids in ferroptosis occurs strictly in endoplasmic reticulum-associated compartments and that only one class of phospholipid—PE molecules with AA or AdA acyl chains—is oxidized [57]. The enzyme lipoxygenase (15-LOX) was found to oxidize AA-PE and AdA-PE molecules to doubly and triply oxygenated-(15-hydroperoxy-) diacylated PE species. A key experiment was that added preformed PE-AA-OOH, but not AA-OOH, enhanced RSL-3-induced ferroptosis in *Ascl4* KO cells, which have low levels of AA-PE lipids [57]. This finding showed that PE-AA-OOH is the molecule that mediates cell death in ferroptosis (Figure 6). Another important finding was that vitamin E inhibits LOX and thereby protects against ferroptosis.

### 4.5. Conclusions

Because of its unique physical properties, PE is at the hub of numerous cellular processes. Recent studies on lipid-induced ER stress and ferroptosis show an intricate balance between saturated and unsaturated lipids in the membranes of the ER. Disruption of this balance can be devastating to cells. An excess of saturated lipids in the ER membranes decreases membrane fluidity and triggers ER stress and the attending response, if left unchecked, leads to cell death. On the other hand, an excess of PE species with polyunsaturated acyl chains in the ER membranes can—if there are any perturbations of redox buffering—trigger the formation of toxic PE hydroperoxides that kill cells. As our knowledge of these pathways deepens, one can expect that drugs will be able to alter the balance between saturated and unsaturated fatty acids to minimize ER stress and to prevent unwanted lipid peroxidation.

That PE as a target of OPA is fascinating. This work will inspire, in our opinion, investigations to explore how PE accumulates on the surface of some cancer cells. Further exploration of the precise mechanism by which OPA-PE adducts kill cells is needed. That LACTB is a tumor suppressor that functions to downregulate PISD, and consequently, PE/LPE is a stunning finding that will open up new areas regarding the role of mitochondrial lipids in metabolism and proliferation.

The role of lipids in neurodegeneration, especially in PD, is a fertile area of research. Given the propensity of *α*-synuclein to bind membranes and vesicles, it is likely that lipid metabolism plays a role in the conversion of this protein from a nontoxic protein into a toxic one. Years of investigations regarding *α*-synuclein still have not uncovered the mechanisms involved in its age-dependent aggregation, how aggregates kill cells, and how to prevent the formation of toxic aggregates in the first place.
